# Cities on the Coast and Patterns of Movement between Population Growth and Diffusion

**DOI:** 10.3390/e23081041

**Published:** 2021-08-13

**Authors:** Dmitry V. Kovalevsky, Dimitri Volchenkov, Jürgen Scheffran

**Affiliations:** 1 Climate Service Center Germany (GERICS), Helmholtz-Zentrum Hereon, Fischertwiete 1, 20095 Hamburg, Germany; 2 Department of Mathematics and Statistics, Texas Tech University, 1108 Memorial Circle, Lubbock, TX 79409, USA; dimitri.volchenkov@ttu.edu; 3 Research Group Climate Change and Security (CLISEC), Institute of Geography, Center for Earth System Research and Sustainability (CEN), Universität Hamburg, Grindelberg 7, 20144 Hamburg, Germany; juergen.scheffran@uni-hamburg.de

**Keywords:** coastal urban dynamics, growth-diffusion models, maximum entropy principle, climate change, climate adaptation

## Abstract

Sea level rise and high-impact coastal hazards due to on-going and projected climate change dramatically affect many coastal urban areas worldwide, including those with the highest urbanization growth rates. To develop tailored coastal climate services that can inform decision makers on climate adaptation in coastal cities, a better understanding and modeling of multifaceted urban dynamics is important. We develop a coastal urban model family, where the population growth and urbanization rates are modeled in the framework of diffusion over the half-bounded and bounded domains, and apply the maximum entropy principle to the latter case. Population density distributions are derived analytically whenever possible. Steady-state wave solutions balancing the width of inhabited coastal zones, with the skewed distributions maximizing population entropy, might be responsible for the coastward migrations outstripping the demographic development of the hinterland. With appropriate modifications of boundary conditions, the developed family of diffusion models can describe coastal urban dynamics affected by climate change.

## 1. Introduction

Coastal zones are some of the most densely populated areas in the world and include the world’s largest cities and the fastest growing urban areas [1,2,3]. If the U.S. coastal counties were an individual country, it would rank third in the world in gross domestic product, surpassed only by the United States as a whole and China [4], producing more than $9.0 trillion in goods and services, employing more than 57.5 million people, and paying $3.6 trillion in wages annually (statistics are for 2016) [5]. The COVID-19 pandemic heightened the debates in urban circles on post-pandemic urban growth strategies and boosting the growth of towns and cities in coastal areas, taking pressure off the capital cities [6].

On-going and projected climate change negatively affects humans, ecosystems and the economy globally and regionally [7,8,9,10,11,12,13] on the emergency scales [14] tailored for use at the appropriate geographic level—city, county, or state—and many of these adverse impacts are particularly pronounced in coastal areas. Many of these areas are increasingly affected by sea level rise and climate-related coastal hazards [15]. Coastal cities are particularly endangered due to the high density of urban populations and the vulnerability of critical infrastructures [16]. With tailored climate services informing regional and local decision making on climate adaptation [17,18,19,20], coastal cities in particular need *coastal climate services* [21,22].

While the provision of climate services crucially depends on state-of-the-art climate science information, the development of tailored products for end-users also implies knowledge co-creation with stakeholders and often requires a profound understanding of regional and local socio-economic dynamics. In the context of coastal climate services for cities, particularly if they are targeted on urban infrastructures, modeling coastal urban dynamics at various spatio-temporal scales can play an important role. Therefore, the present paper is focused on modeling the dynamics of coastal cities.

Depending on the particular research question, socio-natural systems under climate and environmental change, including coastal urban systems, can be studied within very different modeling approaches, including, for example, *system dynamics* (SD) [23,24,25,26,27] and *actor-based system dynamics* [28,29,30], *cellular automata* (CA) [31,32,33,34,35,36,37,38,39,40], *agent-based modeling* (ABM) [41,42,43,44], the *VIABLE* modeling framework [45,46,47], and *optimization models* [48]. Another important group of approaches to modeling urban growth in the coastal zone are aimed at the assessment of multiple factors, such as coastal erosion, pollution and water quality [49], as well as a variety of site attributes essential for the strategic design of Integrated Coastal Zone Management with the use of Artificial Intelligence (AI) methods. For example, the *Land Transformation Model* [50] is based on the available GIS data arrays [51], remote sensing and geospatial analysis, performed by artificial neural networks (ANN) that “learn” about the complex spatial relationships of factors that might correlate with urban development [52,53,54] at different aggregation levels and cell definitions [55,56,57,58]. The dynamic modeling by AI first requires the outlining of future urban growth perspectives in coastal watersheds [59], and then the ANN learns the plausible data traits on urban development from the available datasets to project the data into the future in the course of simulation. Many authors have modeled urban growth using different scenarios of urban changes [31,32,33,34,35,36,41,42,50,60,61,62] on the condition of urban sustainable development at a variety of very diverse sustainability criteria [27,37,38].

All these approaches have also been actively applied to modeling urban dynamics in general and coastal urban dynamics in particular, including impacts of, and response to, climate change [63,64,65,66,67,68,69,70,71,72,73,74]. We review the strengths and weaknesses of these modeling approaches, as applied to urban studies, in Section 2.1 below. The number of urban change models grows rapidly, the employed data aggregation methods are very diverse, and the modeling results obtained by these approaches are often controversial and scenario-sensitive, which makes the decision about what model to use for adaptation planning, policy making, and practice responses difficult.

In the present paper, we apply the *growth-diffusion models* and the *maximum entropy principle* to simulate the dynamics of a coastal city. In contrast to the previously mentioned approaches, we neither employ assumption-dependent numerical simulations, nor play with the pre-selected data. We start from the first principles of mathematical modeling, and write down and solve the differential equations describing the growth-diffusion processes in the presence of a *boundary*.

In our previous work [75,76,77,78,79], we demonstrated that an *extended boundary* cutting the city fabric, such as the coast, a railway or an industrial zone, can create the structurally isolated pockets of streets and other inner-city areas nearby that might foster the physical segregation of minority groups living there and cause their economic marginalization. In our present paper, we model another important effect of the boundary on urban dynamics that may explain the concentration of the population in urban areas close to extended boundaries, for example, in coastal zones. Therefore, extended boundaries can play a dual role in urban dynamics, simultaneously accumulating and structurally barricading people from the mainland regional areas. The proposed family of simple one-dimensional (1D) diffusive models with a boundary can help with understanding the coastal urban dynamics exposed to adverse climate change impacts. The dynamics of the proposed growth-diffusion models can inform adaptation planning, policy making, and practice responses, for all economic activity occurring in coastal areas, including migration and the planned resettlement of exposed vulnerable populations.

In our approach, the urban population density distributions are derived from the solutions of the growth-diffusion partial differential equations (PDE) supplied with the appropriate initial and boundary conditions at the coast, as well as in a band representing a coastal zone. We also apply the maximum entropy principle to the analysis of equilibrium population density.

The statistical tendency of a thermodynamic system to increase its entropy manifests itself in the form of *entropic force* [80,81], transforming the probability distributions over time in a way that adjusts to the *maximum entropy principle* [82,83]. In thermodynamics, the probability distributions maximizing entropy are viewed as the most probable states of the system observed over a very long time. The validity of thermodynamic laws in the description of collective human behavior and population dynamics was verified for the distributions of inhabitants in Spanish provinces, including the tendency for the progressive abandonment of the countryside in favor of the city [84] and for the demographic data on the world population age structures collected over 231 countries and areas over a period of 66 years [85]. Other applications of entropy concepts to geographical modeling are described and reviewed in [86,87,88].

In our work, we implement the maximum entropy principle to predict the critical parameters of urban dynamics, such as the width of the stable inhabited coastal zones and the related skewed stationary population distributions, balancing the drivers of coastal urban dynamics. The proposed method establishes a profound connection between the maximum entropy principle, urban dynamics, and the concentration of the population along sea borders. Our models allow for contriving the global estimates that give a sense of the scale and spatial distribution of the population exposed to rising sea levels, while many other factors are needed for understanding and managing potential impacts and for planning for adaptation locally [3]. We left the detailed assessment of the coastal urban dynamics affected by sea level rise and climate-related coastal hazards for a follow-up paper to be published elsewhere.

The rest of this paper is structured as follows. Section 2 introduces three models of coastal urban dynamics within the framework of diffusion in bounded domains. In Section 3, we study the proposed models and describe the most important features of their solutions. We provide a discussion in Section 4 and then a conclusion in Section 5. Our paper also contains six appendices describing the details of calculations.

## 2. Methods 

### 2.1. Strengths and Weaknesses of Urban Modeling Approaches 

As mentioned above, a number of modeling approaches allow the modeling of the facets of complex (coastal) urban dynamics. Given the complexity of the system under study, these approaches should be seen as complementary: each approach has its own advantages and disadvantages, and none of them is universal.

In particular, CA models integrate mathematical theories of self-reproduction in automata and stochasticity with the two-dimensional cartographic space. A generic concept of a dynamic geographical cellular automata (CA) proposed by Tobler [89] was applied mostly in urban growth and change models. CA as mathematical constructs are known as a standard example of complex systems; in particular, they obey the four key ‘fingerprints of complexity’: emergence, instability, irreducibility and adaptability [90]. At the same time, urban CA have sometimes been criticised for their overcomplexity and a somewhat ‘mechanistic’ viewpoint on land use change processes, as well as for the underdevelopment of their calibration methods (see a discussion in [64]).

ABM is a paradigm where an economy is described as a population of a large number of individual economic agents characterized by bounded rationality. Normally, the population of model agents is evolving in space, that is, the (geographical) space is explicitly accounted for in the modeling framework (with the impressive evolution from stylized geographical spaces in earlier pioneering works to realistic, often GIS-integrated geography in later research). ABM models, being built bottom-up and often based on pretty simple rules and strategies of individual agents, often demonstrate a non-trivial emergent behavior. As the ABM approach accounts for the heterogeneity of actors, ABMs are particularly good candidates for accounting for inequality effects in assessing the dynamics of urban populations. At the same time, ABM models are often difficult to validate against real-world data.

SD is substantially based on a vision of the urban system as being governed by the interactions of a few key aggregated actors. The economy is treated as a nonlinear dynamical system with non-trivial feedback controls. One of the strengths of SD as a method is its ability to model the disequilibrium dynamics. In the context of modeling coastal urban dynamics, this might be particularly relevant for describing, for example, the recovery pathways/adjustments after extreme events [91]. An obvious limitation of SD modeling is that the models of this kind are generally non-spatial and therefore do not sufficiently incorporate the geographical effects.

Optimization models, by definition, find ‘the best’ solution and—in the context of optimal control problems and intertemporal optimization—‘the best’ control strategy, maximizing a certain goal function. At the same time, much attention in the literature on the economics of climate change and integrated assessment modeling has been paid to the sensitivity of intertemporal optimization models to the value of the discount rate, which also raises the question about the ‘right’ value of this fundamental model parameter (see [92] and references therein).

In the present study, we adopt the growth-diffusion modeling approach that shares many advantages with the other approaches briefly discussed above. Like CA and ABM, the growth-diffusion models explicitly incorporate the geographical space. Similarly to ABM, they include (albeit in a simplistic way) agent interactions; similarly to CA, a concept of neighborhood is fundamentally important in defining the model dynamics. The growth-diffusion modeling might be seen, in a way, as spatially-explicit SD models; at the same time, they can, in principle, also incorporate the inter-temporal optimization scheme.

The key assumption of the approaches discussed above is that the observed trends of coastal urban growth, the simplistic behavior rules and strategies of agents and the aggregated actors are likely to continue indefinitely into the future. Our approach is more fundamental and therefore presumably less sensitive to the possible forthcoming regime shifts in the socioeconomic trends of coastal urban systems in the sense that we use the single following assumption in our work: *although the coastline may change notably due to sea level rise, the physical coast will always be profoundly affecting the entire system dynamics through boundary conditions.* The effect of boundary conditions on restricting the available band of wave vectors for possible solutions, which fall below their bulk values near the boundary in the diffusion and reaction–diffusion equations, is well known [93].

### 2.2. Modeling the Coastal Urban Dynamics within the Growth-Diffusion Framework

In our modeling of coastal urban dynamics, we follow a generic framework for combining spatial diffusion with non-spatial growth models presented in a review of spatial diffusion models with applications to case studies from various research domains in geographical space ([64], Section 3.1.1.6).

In the 1D geometric setting, the general growth-diffusion model takes the following form:

(1) $$\frac{\partial N}{\partial t}\left(x,t\right)=f\left(N\left(x,t\right)\right)+D\frac{\partial^{2}N}{\partial x^{2}}\left(x,t\right),$$

where $N\left(x,t\right)$ is the *population density*, *D* is the *diffusion coefficient*, a proportionality constant between the population flux and the gradient in the achieved population density. The generic *non-spatial* population growth model reads as follows:

(2) $$\frac{dN\left(t\right)}{dt}=f\left(N\left(t\right)\right),$$

for some function $f\left(\cdot \right)$. The general Cauchy problem for Equation (1) amounts to determining the appropriate shape of the boundary and the proper initial and boundary conditions, which yield unique and reasonable solutions for the PDE.

A pioneering successful application of a two-dimensional (2D) linear growth-diffusion equation in ecology successfully explained the long-term field data on animal population dynamics in geographical space [94,95]. Closer to the topic of the present study, a 2D nonlinear (logistic) growth-diffusion model was applied to urban population dynamics (with a neighborhood change) in [96].

In the present paper, we propose a 1D coastal urban model family based on a generic 1D growth-diffusion equation of type Equation (1).

**Figure 1:**
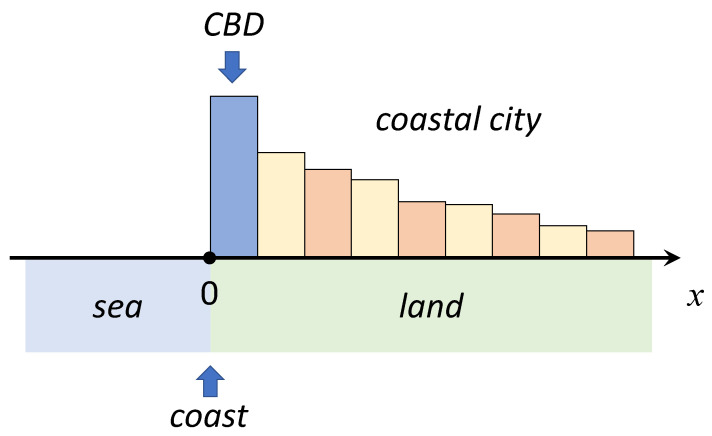
The geometry of a one-dimensional coastal urban model. x=0 corresponds to the coastline, x>0 to the land, and x<0 to the sea. The central business district (CBD) of the city is located at the coast (x=0).

In our model setup (Figure 1), the spatial coordinate *x* specifies the distance from a straight coastline inland in the direction orthogonal to the coastline: x=0 corresponds to the coastline, x>0 to the land, and x<0 to the sea. For certain modifications of the models considered below, we highlight the interpretation of a coastal boundary x=0 as the central business district (CBD) of the city. While most of solutions in the paper are derived for a semi-infinite geometry 0≤x<+∞, in Section 3.2.4 below, we additionally consider the urban area in a finite coastal band 0≤x≤L.

Our interpretation is that $N\left(x,t\right)$ in Equation (1) represents the *population density* in the urban area; alternatively, we might assume that $N\left(x,t\right)$ is the spatial density, or the value of real estate per unit area. Therefore, our model describes a coastal city that is translation invariant along an infinite straight coastline and gradually develops over time from the coastline to further inland (in the future a simplifying assumption of 1D geometry of the problem could be dropped, taking a realistic geography of a city and describing urban sprawl). In this setting, we consider three alternative variants of a non-spatial growth model in Equation (2): the case of no growth (the r.h.s. of Equation (2) is zero) and the cases of exponential and logistic growth, respectively.

For these three models, the general Equation (1) takes the following forms:

- **Model 1:** The *simple diffusion* model, viz.,
  (3) $$\frac{\partial N}{\partial t}=D\frac{\partial^{2}N}{\partial x^{2}}.$$
- **Model 2:** The *exponential growth-diffusion* model, viz.,
  (4) $$\frac{\partial N}{\partial t}=rN+D\frac{\partial^{2}N}{\partial x^{2}},$$
  where *r* is the population growth rate; and, finally,
- **Model 3:** the *logistic growth-diffusion* model, viz.,
  (5) $$\frac{\partial N}{\partial t}=rN\left(1-N\right)+D\frac{\partial^{2}N}{\partial x^{2}}.$$

In the latter model, the following asymptotic growth limit is implied at t→+∞ for the non-spatial logistic growth model (in case the diffusion term in Equation (5) is dropped), N(x,+∞)=1. If it were instead $N\left(x,+\infty \right)=N_{\infty }$, we could rewrite Equation (5) in a generalized form, viz.,

(6) $$\frac{\partial N}{\partial t}=rN\left(1-\frac{N}{N_{\infty }}\right)+D\frac{\partial^{2}N}{\partial x^{2}},$$

where $N_{\infty }$ is the *carrying capacity* of an environment [97] in the classical logistic growth model. By normalizing the density, $N\left(x,t\right)/N_{\infty }$, we return Equation (6) to the logistic growth-diffusion model (5), which we use in the rest of our paper.

Below, we assume the following initial and boundary conditions for all three models, including zero population density everywhere at t=0, before the city emerges, viz.,

(7) $$N\left(x,0\right)=0,\;x\geq 0.$$

As the boundary condition at the coast (x=0), in all but one simulation we assume a step-wise increase of the population density from zero to a certain positive value $N_{0}$ at t=0, subsequently maintaining the density at the same constant level at all future time moments (which describes the city emerging at the coast and propagating further inland, under the assumption of continuous migration to the coastal city from overseas maintaining the boundary condition at the coast):

(8) $$N\left(0,0\right)=0;\;N\left(0,t\right)=N_{0}\;\;\mathrm{for}\;\;t>0.$$

The boundary condition at x=+∞ is assumed to be trivial, viz.,

(9) $$N\left(x,t\right)\to 0\;\;\mathrm{at}\;\;x\to +\infty .$$

For the exponential growth-diffusion model, we will additionally assume a time-dependent boundary condition at the coast (see Section 3.2 below).

We now discuss the numerical values of model parameters chosen by us for exemplary simulations. When studying urban population dynamics in the proposed setting, it is natural to choose kilometers and years as spatial and temporal units, respectively. Then, for 1D diffusion problems, the characteristic length scale is $x^{2}∼2Dt$. Indeed, if we were considering a diffusion over the entire real axis −∞<x<+∞, not over the positive semi-axis x≥0 only, then the initial localized density perturbation $N\left(x,0\right)=\delta \left(0\right)$ would evolve in 1D Gaussian distribution of the form [98]

(10) $$N\left(x,t\right)=\frac{1}{\sqrt{4\pi \cdot Dt}}\exp\left(-\frac{x^{2}}{4\cdot Dt}\right),$$

with a variance

(11) $$\sigma^{2}=2Dt.$$

Assuming that the city spreads for 10 km in 50 years, we get from Equation (11) an estimate $D=1\;{\mathrm{km}}^{2}/\mathrm{year}$—the value we will be using in our simulations for all three models. We also choose $r=0.01\;{\mathrm{year}}^{-1}$ (i.e., 1% per year) for the second and the third models—a value not uncommon in many economic or demographic processes.

Regarding the units for population density, as follows from the discussion above, we can assume that it is a non-dimensional field: both for the first two models that are linear, and for the third, nonlinear, model assigning a dimension to density would be just a matter of proper normalization. After specifying the time-independent boundary condition $N_{0}$ at the coast for the first two models, $N_{0}$ merely appears as a factor in the derived solutions, due to the linearity of these models, so that the properties of these solutions do not depend on the specific value of $N_{0}$.

For the third (nonlinear) model, a particular case $N_{0}=1$ yields the simplest possible solution, as unity is also the limit of the logistic growth (the carrying capacity) in Equation (5). Additionally, we will consider values for $N_{0}$ greater or less than unity for the third model.

## 3. Results 

### 3.1. Model 1: The Simple Diffusion Model

The *simple diffusion* model of coastal urban dynamics is fully specified by Equation (3), initial conditions (7) and boundary conditions (8) and (9). The problem under consideration allows the exact analytical solution [98], viz.,

(12) $$N\left(x,t\right)=N_{0}\left[1-\mathrm{erf}\left(\frac{x}{2\sqrt{Dt}}\right)\right],$$

or, equivalently,

(13) $$N\left(x,t\right)=N_{0}\;\mathrm{erfc}\left(\frac{x}{2\sqrt{Dt}}\right),$$

where the *error function* is defined as

(14) $$\mathrm{erf}\left(y\right)=\frac{2}{\sqrt{\pi }}\int_{0}^{y}\exp\left(-z^{2}\right)dz,$$

and the *complementary error function* is defined as

(15) $$\mathrm{erfc}\left(y\right)=\frac{2}{\sqrt{\pi }}\int_{y}^{+\infty }\exp\left(-z^{2}\right)dz.$$

**Figure 2:**
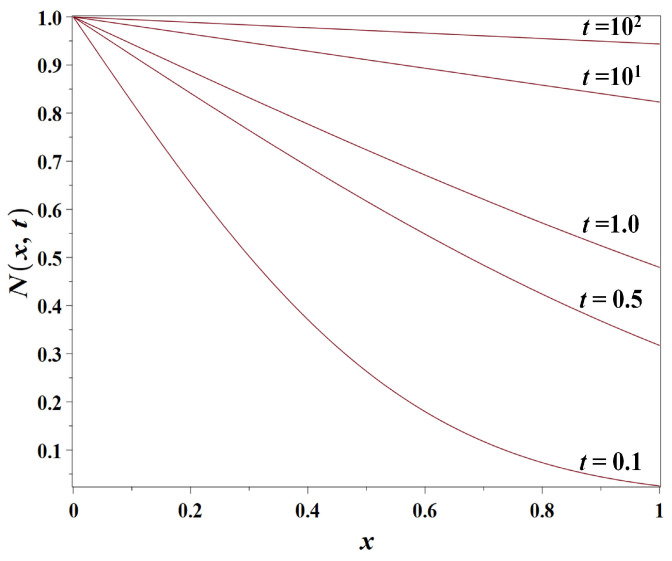
Snapshots of the solution of Model 1 (non-dimensional population density N(x,t) as simulated by the simple diffusion model (3)) for the population diffusion coefficient $D=1\;{\mathrm{km}}^{2}/\mathrm{year}$, $N_{0}=1$, at t=0.1, t=0.5, t=1.0, t=10 and t=100 years, respectively. The distance from the coast *x* is measured in km.

This solution (13) describes the population diffusion from the coastal zone inland (see Figure 2).

The density is controlled by the boundary condition maintained at the coast $N_{0}=1$.

Knowledge of population density $N\left(x,t\right)$ allows us to compute some aggregate characteristics of urban population dynamics, such as the total urban population $N_{*}\left(t\right)$,

(16) $$N_{*}\left(t\right)=N_{0}\frac{2}{\sqrt{\pi }}\sqrt{Dt}$$

(see Appendix A). Moreover, dividing the value $N\left(x,t\right)$ by the total population $N_{*}\left(t\right)$, we obtain the probability density function (PDF) $p\left(x,t\right)$ to choose the living location *x* at time *t* as follows:

(17) $$p\left(x,t\right)=\frac{1}{\sqrt{Dt}}\int_{x/2\sqrt{Dt}}^{+\infty }\exp\left(-y^{2}\right)dy.$$

Based on Equation (17), it can further be shown (see Appendix B) that the expected living location of a city inhabitant that characterizes the mean extension of the city growing inland, viz., $\bar{x}=\int_{0}^{+\infty }xp\left(x,t\right)dx$, is then equal to

(18) $$\bar{x}=\frac{\sqrt{\pi }}{2}\sqrt{Dt},$$

and the second moment, $\bar{\;x^{2}\;}=\int_{0}^{+\infty }x^{2}p\left(x,t\right)dx,$ is equal to

(19) $$\bar{x^{2}}=\frac{4}{3}Dt,$$

so that variance quantifying how far the living locations are spread out from their average value Equation (18), marking the gradual *spread of inhabited area* from the coastal region inland, is given by

(20) $$\sigma_{x}^{2}\left(t\right)\;\;=\;\;\left(\frac{4}{3}-\frac{\pi }{4}\right)Dt\;\;\approx 0.55\;\;Dt,$$

and therefore the estimated 0.55D≈0.55 km${}^{2}$/year represents the *average spread rate* of the city inland from the coastal zone in Model 1 (Equation (3)). The standard deviation, measuring the amount of dispersion of the inhabited locations in Model 1 with time, $\sigma_{x}\left(t\right)\propto \sqrt{Dt},$ grows the same way as the total urban population $N_{*}\left(t\right)$ (Equation (16)). Note that, up to a numerical factor, Equations (18)–(20) are in agreement with simple scaling arguments used at the end of Section 2 above to estimate the plausible value for the diffusion coefficient *D*.

### 3.2. Model 2: The Exponential Growth-Diffusion Model

#### 3.2.1. Reduction to the Simple Diffusion Model

The *exponential growth-diffusion* model (4) is supplemented with the initial conditions (7) and boundary conditions (8) and (9). We will consider two alternative model setups, with time-dependent and time-independent boundary conditions at the coast, respectively.

To solve the PDE (Equation (4)), we define an auxiliary ‘tilded’ density filed $\tilde{N}\left(x,t\right)$ such that

(21) $$N\left(x,t\right)=\exp\left(rt\right)\tilde{N}\left(x,t\right).$$

Substituting $N\left(x,t\right)$ in the form (21) in Equation (4), we easily find that the ‘tilded’ density obeys the conventional diffusion Equation (3) considered above,

(22) $$\frac{\partial \tilde{N}}{\partial t}=D\frac{\partial^{2}\tilde{N}}{\partial x^{2}},$$

so that the results derived above in Section 3.1 stay valid, provided the boundary conditions at the coast for the ‘tilded’ density remain the same as for the ‘untilded’ density in the previous model. We discuss this case in more detail in Section 3.2.2.

#### 3.2.2. ‘Synchronized’ Boundary Condition

In the setup for the exponential growth-diffusion model, we first assume the ‘synchronized’ boundary condition for the ‘untilded’ population density at the coast of the following form:

(23) $$N\left(0,0\right)=0;\;N\left(0,t\right)=N_{0}\exp\left(rt\right)\;\;\mathrm{for}\;\;t>0.$$

**Figure 3:**
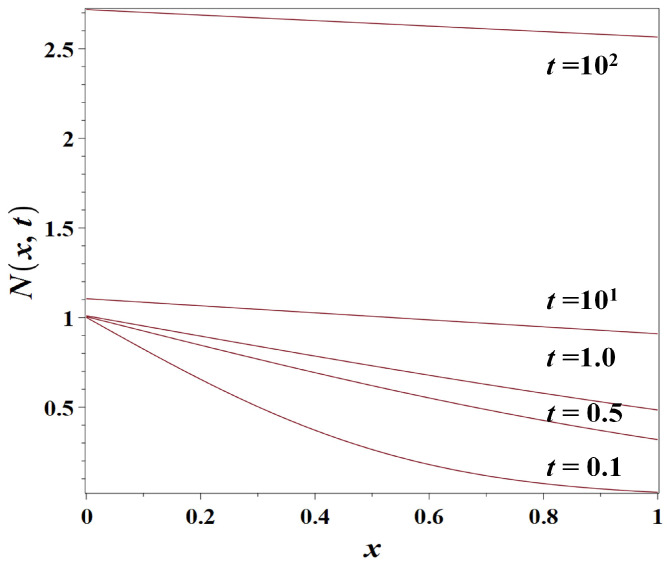
Snapshots of the solution of Model 2 (non-dimensional population density N(x,t) as simulated by the exponential growth-diffusion model (4)) on the synchronized boundary conditions (23), for the population diffusion coefficient $D=1\;{\mathrm{km}}^{2}/\mathrm{year}$, the growth rate $r=0.01\;{\mathrm{year}}^{-1}$, $N_{0}=1.0$, at t=0.1, t=0.5, t=1.0, t=10, and t=100 years, respectively. The distance from the coast *x* is measured in *km*.

As discussed in Section 3.2.1, this means that for the ‘tilded’ population density, we arrive at the simple diffusion model (Model 1) (Equation (3)) discussed in Section 3.1. Therefore, the solution for the ‘untilded’ density with the boundary condition (23) (see Figure 3) coincides with the solution for the previous model (13) up to the growing exponent factor, $\exp\left(rt\right)$, viz.,

(24) $$N\left(x,t\right)=N_{0}\exp\left(rt\right)\;\mathrm{erfc}\left(\frac{x}{2\sqrt{Dt}}\right).$$

As might be expected, the total urban population $N_{*}\left(t\right)$, scales the same way as in Model 1 (Equation (16)), up to the exponential factor, viz.,

(25) $$N_{*}\left(t\right)=N_{0}\frac{2}{\sqrt{\pi }}\sqrt{Dt}\exp\left(rt\right).$$

However, the results (18)–(20) for the spread of inhabited areas from the coastal region inland remain unchanged.

#### 3.2.3. Constraining the Growth in the Central Business District

In the previous setup, the population density exhibited exponential growth Equation (23) uniformly at every location *x*, including the coast (x=0). We now consider an alternative setup for the exponential growth-diffusion model (4), where the density at the coast is maintained at the constant level. Therefore, we return to the boundary condition (8) with $N\left(0,t\right)=N_{0}=\mathrm{const}$. This model setup might be interpreted as the case of imposed *regulations on the population density* in the central business district (CBD) of the city associated with its coastal zone.

This means that, for the ’tilded’ density (see Equation (21) above), we now have a ‘decaying’ boundary condition of the form

(26) $$\tilde{N}\left(0,0\right)=0;\;\tilde{N}\left(0,t\right)=N_{0}\exp\left(-rt\right)\;\;\mathrm{at}\;\;t>0.$$

The solution $\tilde{N}\left(x,t\right)$ of an auxiliary problem Equation (22) with the boundary condition (26) can be found from the solution (13) obtained for the conventional diffusion model by applying *Duhamel’s principle* (see Appendix C). We can return to the ‘untilded’ density afterwards.

Let us define a *characteristic length scale* $x_{G}$ of the exponential growth-diffusion problem:

(27) $$x_{G}=\sqrt{\frac{D}{r}}.$$

Then, the solution for the exponential growth-diffusion model Equation (4) with the boundary condition (8) is given by

(28) $$N\left(x,t\right)=\frac{N_{0}}{2\sqrt{\pi }}\frac{x}{x_{G}}F\left(rt,\frac{x}{x_{G}}\right),$$

where a new function $F\left(\cdot ,\cdot \right)$ is defined as

(29) $$F\left(a,b\right)=\int_{0}^{a}w^{-3/2}\exp\left(w-\frac{b^{2}}{4w}\right)dw.$$

**Figure 4:**
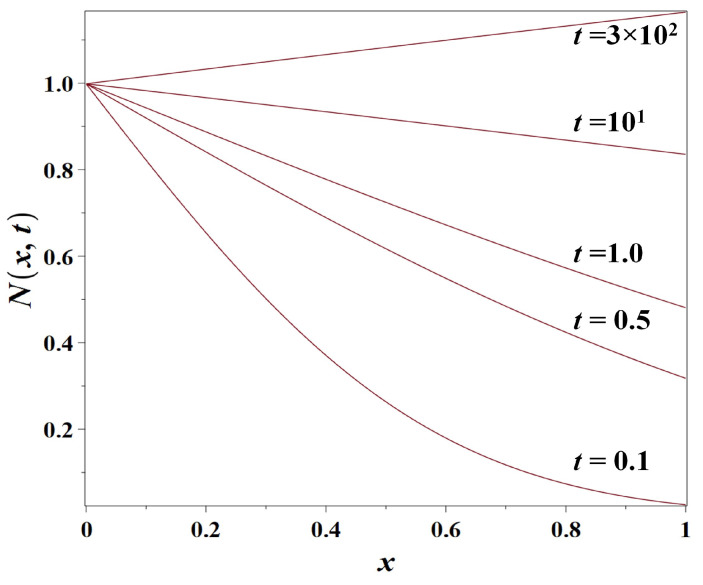
Snapshots of the solution of Model 2 (non-dimensional population density N(x,t) as simulated by the exponential growth-diffusion model (4)) if the population density at the coast is maintained at a constant level, for the population diffusion coefficient $D=1\;{\mathrm{km}}^{2}/\mathrm{year}$, the growth rate $r=0.01\;{\mathrm{year}}^{-1}$, $N_{0}=1$, at t=0.1, t=0.5, t=1.0, t=10, and t=300 years, respectively. The distance from the coast *x* is measured in km.

At a finite value of the parameter *a*, the integral in Equation (29) is finite as well (there is no singularity at w=0). However, if a→+∞, the integral in Equation (29) diverges. This means that, when t→+∞, the solution (28) grows to infinity (except in CBD, at x=0). So, the imposed regulation in CBD does not prevent an infinite growth of density outside CBD (see Figure 4).

The total urban population under the latter scenario is given by (Appendix D)

(30) $$N_{*}\left(t\right)=\frac{N_{0}x_{G}}{\sqrt{\pi }}\int_{0}^{rt}w^{-1/2}\exp\left(w\right)dw.$$

#### 3.2.4. Steady-State Wave Solutions in a Coastal Band

It is easy to check that the exponential growth-diffusion model (4) allows for the following steady-state wave solution in a band of width L>0:

(31) $$N\left(x,t;L\right)=\frac{B\sin\left(\frac{x}{x_{G}}\right)+A\sin\left(\frac{L-x}{x_{G}}\right)}{\sin\left(\frac{L}{x_{G}}\right)},\;\;0\leq \frac{x}{x_{G}}<\frac{L}{x_{G}}\leq \pi ,$$

if supplied with the initial conditions (at t=0) along the coast (x=0) and at the coastal zone boundary (x=L):

(32) N(0,0)=A;N(L,0)=B.

This steady-state solution (31) corresponds to the stationary expression for the total urban population $N_{*}\left(L\right)$ in the coastal zone,

(33) $$N_{*}\left(L\right)=\int_{0}^{L}\;N\left(x,t;L\right)dx=x_{G}\left(A+B\right)\frac{\left(1-\cos\left(\frac{L}{x_{G}}\right)\right)}{\sin\left(\frac{L}{x_{G}}\right)},$$

and the following stationary probability distribution p(x;L) to find an inhabitant at the location $0<x<L\leq \pi \;x_{G}$ in the coastal band,

(34) $$p\left(x;L\right)=\frac{B\sin\left(\frac{x}{x_{G}}\right)+A\sin\left(\frac{L-x}{x_{G}}\right)}{x_{G}\left(A+B\right)\left(1-\cos\left(\frac{L}{x_{G}}\right)\right)},$$

characterized by the band *population entropy* functional,

(35) $$H\left(L\right)=-\int_{0}^{L}p\left(x;L\right)\ln p\left(x;L\right)dx.$$

The latter functional attains its maximum value for x>0 when

(36) $$\begin{array}{ccc} 0=\frac{dH(L)}{dL} & = & -p(L;L)\ln p(L;L)\\ \; & \; & =-\frac{C\sin\left(x/x_{G}\right)}{1-\cos\left(L/x_{G}\right)}\ln\left(\frac{C\sin\left(x/x_{G}\right)}{1-\cos\left(L/x_{G}\right)}\right),\;\;C\equiv x_{G}\frac{B}{A+B}, \end{array}$$

that is, when the width of the populated coastal band takes the value

(37) $$L_{*}\left(x_{G};C\right)=x_{G}\;\arctan\left(\frac{2C}{C^{2}+1},\frac{1-C^{2}}{C^{2}+1}\right)\leq \pi \;x_{G},$$

where arctan(x,y) is the angle in the Euclidean plane, between the positive *x*-axis and the ray to the point (x,y), that is,

(38) $$\arctan\left(x,y\right)\equiv \mathrm{sgn}{\left(x\right)}^{2}\arctan\left(\frac{y}{x}\right)+\frac{\pi \left(1-\mathrm{sgn}(x)\right)}{2}\left(1+\mathrm{sgn}\left(y\right)-\mathrm{sgn}{\left(y\right)}^{2}\right).$$

**Figure 5:**
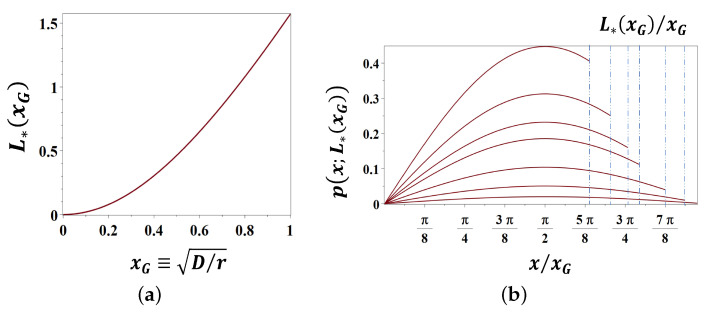
(**a**) The width of the populated coastal band $L_{*}$ providing the maximum entropy for the population density as the function of the characteristic length scale $x_{G}\equiv \sqrt{D/r}$. (**b**) The stationary distributions $p\left(x;L_{*}\left(x_{G}\right)\right)$ maximizing the entropy functional for the different values of the characteristic scale $x_{G}$ are shown in the respective intervals $0\leq x/x_{G}<L_{*}\left(x_{G}\right)/x_{G}\leq \pi$.

Let us assume that A≡N(0,0)=0, then, for any B>0, we get $C=x_{G}$ in Equation (36), and therefore $L_{*}$ (Equation (37)) appears to be a function of the characteristic length scale $x_{G}\equiv \sqrt{D/r}$ alone (see Figure 5a).

The skewed stationary probability distributions $p\left(x;L_{*}\left(x_{G}\right)\right)$, maximizing the entropy functional Equation (35) for the the different values of the characteristic scale $x_{G}$, are shown in Figure 5b.

The tendency to the transformation of a thermodynamic system in the direction that increases its entropy is known as the *maximum entropy principle* [82,83], and the forces emerging from the mutual influence of open systems on each other, changing into more probable states of higher entropy, are known as the *entropic forces* [80,81]. In the present subsection, we have shown that the superposition of the diffusion and exponential growth processes in a band might give rise to the steady-state wave solutions of Equation (4) maximizing the entropy functional Equation (35). The action of entropic forces on the population distributions, emerging from the statistical tendency of the system to increase its entropy, results in the formation of the skewed stationary distributions of inhabitants in the urban coastal zone (Figure 5b) specified by the characteristic scale $x_{G}$ and persistent over a very long time. Once a city is built, its physical form and land use patterns, represented in our models by the parameter $x_{G}$, can be locked in for generations. Maintaining the population distribution in the coastal zone steady at traditionally lower urban fertility would require the progressive population migration into urban coastal areas and escalating abandonment of the countryside, as discussed in [84].

### 3.3. Model 3: The Logistic Growth-Diffusion Model

Finally, we consider the *logistic growth-diffusion* model (5) supplied by the initial condition (7) and boundary conditions (8) and (9). A logistic growth-diffusion urban model in two dimensions and without boundary conditions related to the coastal zone was considered in [96].

No exact analytical solution is possible in this case for the time-dependent logistic growth-diffusion problem. However, what is possible here is to derive an analytical solution $N_{\infty }\left(x\right)$ for the stationary case, to which the population density converges at large time (t→+∞). This stationary solution differs from the obvious particular stationary case $N_{\infty }\left(x\right)=1$ if the boundary condition at x=0 is in disagreement with the ‘logistic growth limit’ (the carrying capacity), that is, $N_{0}\neq 1$.

Suppose, for example, that in the boundary condition (8) $0<N_{0}<1$. Like in the model considered in the previous subsection, this can be interpreted as regulations on the population density in the CBD coinciding with the coastal urban zone.

Then the stationary solution is (Appendix E)

(39) $$N_{\infty }\left(x\right)=3{\left(\frac{\exp\left(x/x_{G}\right)-K_{0}}{\exp\left(x/x_{G}\right)+K_{0}}\right)}^{2}-2,$$

where an auxiliary constant $K_{0}$ is defined as

(40) $$K_{0}=\frac{\sqrt{3}-\sqrt{2+N_{0}}}{\sqrt{3}+\sqrt{2+N_{0}}},$$

and, as above, the characteristic length scale $x_{G}$ of the growth-diffusion problem is given by Equation (27).

Equation (39) can also be equivalently re-written to the form

(41) $$N_{\infty }\left(x\right)=3{\left(\frac{1-K_{0}\exp\left(-x/x_{G}\right)}{1+K_{0}\exp\left(-x/x_{G}\right)}\right)}^{2}-2.$$

If the boundary condition is close to unity, $N_{0}≃1$, that is, the CBD restrictions are not tight, as compared to the ‘natural’ carrying capacity limit, then the solution (39) simplifies and can be approximated as (Appendix F)

(42) $$N_{\infty }\left(x\right)≃1-\left(1-N_{0}\right)\exp\left(-x/x_{G}\right).$$

This means that the population density profile has a transitional zone of length scale $x_{G}$ near the coast, but further inland the population density is virtually equal to its carrying capacity value. Note that the asymptotic Equation (42) holds for the values of $N_{0}$ both somewhat less and somewhat greater than unity: the latter case would correspond to the opposite case of (moderately) stimulated construction in the coastal zone.

**Figure 6:**
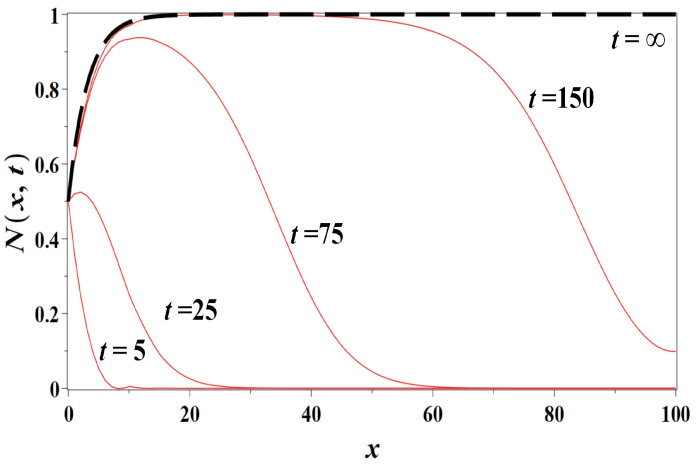
The numeric solutions of Model 3 (non-dimensional population density N(x,t) as simulated by the logistic growth-diffusion model (5)) for the population diffusion coefficient $D=1\;{\mathrm{km}}^{2}/\mathrm{year}$, the growth rate $r=0.01\;{\mathrm{year}}^{-1}$, $N_{0}=0.5$, at t=0.5, t=5, t=25, t=75, t=150 years, along with the stationary solution t=∞ approximated by Equation (42), respectively. The distance from the coast *x* is measured in km.

In Figure 6, we presented the numeric solutions for Model 3 (Equation (5)) at sequential times and the stationary solution for Equation (5) approximated by Equation (42) (t=∞) indicated by the dashed line. The Cauchy problem for the numeric solution of the partial differential Equation (5) has been supplied with the following *Neumann boundary condition*:

(43) $${\left.\frac{\partial N}{\partial x}\left(x,t\right)\right|}_{x=100}=0,$$

which describes a constant population flux from inland areas across the boundary into the coastal zone. The resulting urban coastal dynamics is a growing population wave propagating from the coastal area outward.

## 4. Discussion 

In our work, we have suggested and studied a one-dimensional family of diffusion models with and without growth defined in the half-space with a boundary and in the coastal band. Our approach is based on the analysis of solutions of partial differential equations governing the population density N(x,t) for the case-relevant initial and boundary conditions.

Model 1 (Equation (3)) describes the population diffusion from the coastal zones into inland areas under the condition that the population density is maintained at a constant on the coast (i.e., at the domain boundary). In this model, the total urban population grows proportionally to the square root of time, $\propto \sqrt{Dt},$ in the same way as the variance related to the population distribution characterizing the gradual spread of the inhabited area inland. The solutions are featured by the single diffusion coefficient D.

In Model 2 (Equation (4)), we explored the superposition of diffusion and exponential growth processes, with time-dependent and time-independent boundary conditions on the coast. Two dimensional parameters, *D* (for diffusion) and *r* (for the exponential growth), are combined into the single characteristic length scale $x_{G}=\sqrt{D/r},$ featuring the coastal urban dynamics in the second model. In particular, the superposition of the diffusion and exponential growth processes in the band (of the width $L_{*}≃x_{G}$) might result in the steady-state wave solutions maximizing the entropy functional. The corresponding skewed stationary population distributions anchored in the coastal band and persisting over a long time might explain the progressive population migrations into the urban coastal areas from mainlands.

Finally, in Model 3, we studied the logistic growth-diffusion model (5), in which the population density profile has a transitional zone of length scale $x_{G}$ near the coast, but equals the carrying capacity of the habitat far from the coast.

**Figure 7:**
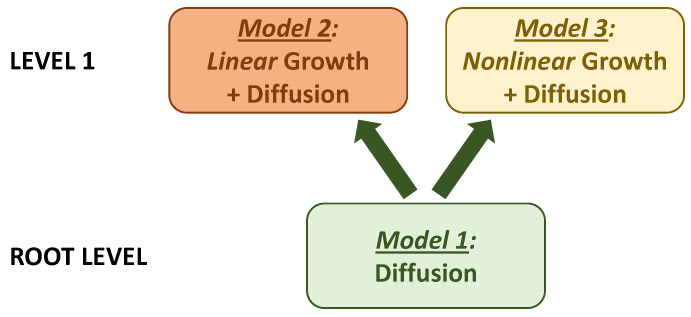
Coastal urban model family organized as a two-level model hierarchy.

As suggested, for example, in [29,99], the process of modeling complex socio-natural systems, to which coastal urban systems definitely belong, can be organized as building model hierarchies. A hierarchy starts at the lowest (root) level with a minimal model. Once the behavior and properties of this root model, as well as its limitations, are well explored and understood, more complexity is added to the model, which results in one or several more complex models at the next hierarchical level. Then the process is repeated, and the model tree grows up, until the models reach the level of complexity where they can no longer be calibrated with, or validated against, the available data. From this perspective, the coastal urban model family developed in the present paper can be seen as a simple two-level model hierarchy (Figure 7). The diffusion model (Model 1) then plays the role of the root model, while adding the growth effects brings the model complexity to the next level (level 1). Two branches of the development of the root diffusion model lead to either the linear or nonlinear growth-diffusion model, respectively.

We see that more detailed growth-diffusion models, placed on the upper level of our small hierarchy, indeed demonstrate more complex dynamics than the root diffusion model. We can also observe that moving up through the levels of complexity does not necessarily mean merely adding second-, third-, …, *n*-th-order effects with gradual ‘convergence’ to ‘the right’ solution. Instead, when going from lower to upper hierarchical levels, model dynamics can change qualitatively. With regard to our coastal urban hierarchy, this is the case, in particular, with respect to the extent of the influence of the boundary condition imposed at the coast. For the root (diffusion) model (Model 1), its influence is more fundamental: the ‘urban wave’ spreads from the coast to further inland, and, after a sufficiently long time, the state of the urban system at any distance from the coast reproduces the condition on the coast. For the next-level growth-diffusion models, growth effects dominate; even when the boundary condition on the coast constrains the growth, its impact is most substantial in the coastal transition zone, while further inland either constrained (Model 3) or unconstrained (Model 2) growth forces take over.

## 5. Conclusions and Outlook 

Populations in coastal zones are growing faster than those in non-coastal areas according to data surveys, especially in the U.S. where approximately 461 people per square mile live in coastal counties [5]. Many of the world’s coasts are becoming increasingly urban [100]. Understanding the processes underlying human migrations and coastal urban dynamics is important in view of adaptation planning and resettlement of the populations potentially exposed to the damaging impacts of rising sea levels and climate-related coastal hazards.

The proposed coastal urban model family can serve as a basis for ‘what-if’ simulations of coastal urban dynamics under climate change. Certain modifications and extensions for this purpose should be brought to the developed growth-diffusion models, with regard to the boundary conditions on the coast. Indeed, in all the models considered above, the coastal boundary condition was static. This would correspond to the situation of a coastal urban system embedded and evolving within a static environment. On-going and projected climate change, as well as other global change factors, in fact, make this environment dynamically evolving, as well. In particular, the effect of sea-level rise and climate-related coastal hazards should be accounted for in the proposed modeling scheme with an appropriate dynamical (time-dependent) boundary condition on the coast. It should be stressed that nothing technically prevents considering the proposed models with time-dependent boundary conditions: in fact, we were already doing this in Section 3.2.3 for the auxiliary (‘tilded’) model.

The proposed models would help to contrive the global estimate of the spatial distribution of the population and urban dynamics in the coastal areas. Further research is required to understand the impact of sea level rise and climate-related coastal hazards on the urban coastal dynamics to improve impact assessments and develop adaptive responses informed by coastal climate services to build sustainable coastal agglomerations. One of the directions of further model development would be describing the tendency of urban populations to move away from risky areas within the gradient approach, by combining the existing physical models and the urban diffusion model with the VIABLE modeling framework [45,46,47].

## Data Availability

Data is contained within the article.

## Abbreviations

The following abbreviations are used in this manuscript:

1D	one-dimensional
2D	two-dimensional
ABM	agent-based modeling
AI	artificial intelligence
ANN	artificial neural network
CA	cellular automata
CBD	central business district
GIS	geographic information system
ODE	ordinary differential equation
PDE	partial differential equation
PDF	probability density function
SD	system dynamics

## Appendix A. Derivation of Total Urban Population for the Simple Diffusion Model (Model 1)

To derive the total urban population for Model 1, we write the solution (13) in integral form by using Equation (15):

(A1) $$N\left(x,t\right)=\frac{2}{\sqrt{\pi }}N_{0}\int_{x/2\sqrt{Dt}}^{+\infty }\exp\left(-y^{2}\right)dy.$$

Then the total urban population

(A2) $$N_{0}\left(t\right)=\int_{0}^{+\infty }N\left(x,t\right)dx$$

is given by

(A3) $$N\left(x,t\right)=\frac{2}{\sqrt{\pi }}N_{0}\int_{0}^{+\infty }dx\int_{x/2\sqrt{Dt}}^{+\infty }\exp\left(-y^{2}\right)dy,$$

or, changing the order of integration,

(A4) $$N\left(x,t\right)=\frac{2}{\sqrt{\pi }}N_{0}\cdot 2\sqrt{Dt}\int_{0}^{+\infty }y\exp\left(-y^{2}\right)dy,$$

from which we easily arrive at Equation (16).

## Appendix B. Derivation of the Expected Living Location and the Second Moment for the Simple Diffusion Model (Model 1)

Firstly, based on a PDF $p\left(x,t\right)$ provided by Equation (17), we calculate the *n*-th moment

(A5) $$\bar{x^{n}}=\int_{0}^{+\infty }x^{n}p\left(x,t\right)dx,$$

or, explicitly,

(A6) $$\bar{x^{n}}=\frac{1}{\sqrt{Dt}}\int_{0}^{+\infty }x^{n}\cdot dx\int_{x/2\sqrt{Dt}}^{+\infty }\exp\left(-y^{2}\right)dy.$$

Changing the order of integration, like in Appendix A, we get:

(A7) $$\bar{x^{n}}=\frac{2^{n+1}}{n+1}{\left(Dt\right)}^{n/2}\int_{0}^{+\infty }y^{n+1}\exp\left(-y^{2}\right)dy.$$

By choosing $y^{2}$ as a new integration variable, the integral in the r.h.s. of Equation (A7) is reduced to $\Gamma \left(\frac{n}{2}+1\right)/2$, where the Gamma function is used,

(A8) $$\Gamma \left(u\right)=\int_{0}^{+\infty }\exp\left(-t\right)t^{u-1}dt.$$

This yields

(A9) $$\bar{x^{n}}=\frac{2^{n}}{n+1}\Gamma \left(\frac{n}{2}+1\right){\left(Dt\right)}^{n/2}.$$

As $\Gamma \left(\frac{3}{2}\right)=\sqrt{\pi }/2$ and $\Gamma \left(2\right)=1$, the general result Equation (A9) yields Equations (18) and (19) in particular cases n=1 and n=2, respectively.

## Appendix C. Derivation of Solution for the Exponential Growth-Diffusion Model (Model 2, Case of Regulated Central Business District)

Consider a problem

(A10) $$\frac{\partial u}{\partial t}=D\frac{\partial^{2}\;u}{\partial x^{2}},$$

(A11) $$u\left(x,0\right)=0,\;x\geq 0,$$

(A12) $$u\left(0,0\right)=0;\;u\left(0,t\right)=\mu \left(t\right)\;\;\mathrm{at}\;\;t>0.$$

If we know the solution $v\left(x,t\right)$ of another problem

(A13) $$\frac{\partial v}{\partial t}=D\frac{\partial^{2}v}{\partial x^{2}},$$

(A14) $$v\left(x,0\right)=0,\;x\geq 0,$$

(A15) $$v\left(0,0\right)=0;\;v\left(0,t\right)=1\;\;\mathrm{at}\;\;t>0,$$

then, according to *Duhamel’s principle*, the solution $u\left(x,t\right)$ of the problem (A10)–(A12) can be found as

(A16) $$u\left(x,t\right)=\int_{0}^{t}\mu \left(s\right)\frac{\partial }{\partial t}v\left(t-s,x\right)ds.$$

The problem (A13)–(A15) is equivalent to the conventional diffusion model considered in Section 3.1. Therefore, in accordance with Equation (13), its solution is

(A17) $$v\left(x,t\right)=\mathrm{erfc}\left(\frac{x}{2\sqrt{Dt}}\right).$$

The problem (A10)–(A12) correspond to the auxiliary problem for the ‘tilded’ density $\tilde{N}\left(x,t\right)$ considered in Section 3.2 if we specify in Equation (A12)

(A18) $$\mu \left(t\right)=N_{0}\exp\left(-rt\right).$$

Then, from Equation (A16),

(A19) $$\tilde{N}\left(x,t\right)=\frac{N_{0}x}{2\sqrt{\pi D}}\int_{0}^{t}\frac{\exp\left(-rs\right)}{{\left(t-s\right)}^{3/2}}\exp\left(-\frac{x^{2}}{4D\left(t-s\right)}\right)ds$$

(see also [98]). By introducing the characteristic length scale $x_{G}$ according to Equation (27) and a new integration variable $\tau =r\left(t-s\right)$, we easily obtain from Equation (A19)

(A20) $$\tilde{N}\left(x,t\right)=\frac{N_{0}}{2\sqrt{\pi }}\frac{x}{x_{G}}exp\left(-rt\right)F\left(rt,\frac{x}{x_{G}}\right),$$

where a new function $F\left(\cdot ,\cdot \right)$ is defined by Equation (29). By returning to ’untilded’ variables, we obtain Equation (28).

## Appendix D. Total Urban Population for the Exponential Growth-Diffusion Model (Model 2, Case of Regulated Central Business District)

We calculate

(A21) $$N_{*}\left(t\right)=\int_{0}^{+\infty }N\left(x,t\right)dx,$$

by substituting the solution (28) and making use of definition (29). Explicitly,

(A22) $$N_{*}\left(t\right)=\frac{N_{0}}{2\sqrt{\pi }x_{G}}\int_{0}^{rt}w^{-3/2}\exp\left(w\right)dw\int_{0}^{+\infty }x\exp\left(-\frac{1}{4w}{\left(\frac{x}{x_{G}}\right)}^{2}\right)dx.$$

The inner integral in Equation (A22) is equal to $2wx_{G}^{2}$, and we arrive at Equation (30).

## Appendix E. Derivation of the Stationary Solution in the Logistic Growth-Diffusion Model (Model 3)

The stationary solution $N_{\infty }\left(x\right)$ of the problem (5) with the initial condition (7) and boundary conditions (8) and (9) is given by a solution of a boundary value problem for the ordinary differential equation (ODE)

(A23) $$n^{\prime \prime }+\frac{1}{x_{G}^{2}}n\left(1-n\right)=0,$$

(A24) $$n\left(0\right)=n_{0},$$

(A25) $$n\left(+\infty \right)=1.$$

In Equation (A23), a prime denotes a derivative over *x*, and a characteristic length scale $x_{G}$ of the growth-diffusion problem is defined by Equation (27). The second-order ODE (Equation (A23)) does not contain an independent variable, so its order can be lowered [98].

Define $p=n^{\prime }$, then $n^{\prime \prime }=p\frac{dp}{dn}$, and variables in Equation (A23) can be separated:

(A26) $$p\;dp=-\frac{1}{x_{G}^{2}}n\left(1-n\right)dn.$$

By integrating Equation (A26), we get:

(A27) $$\frac{p^{2}}{2}=C-\frac{1}{x_{G}^{2}}\left(\frac{n^{2}}{2}-\frac{n^{3}}{3}\right).$$

A constant *C* in Equation (A27) can be determined from a condition at x=+∞, where n=1 and p=0. This yields

(A28) $$C=\frac{1}{6x_{G}^{2}}.$$

Then, from Equations (A27) and (A28),

(A29) $$p=\pm \frac{1}{\sqrt{3}x_{G}}\sqrt{1-3n^{2}+2n^{3}}.$$

As we consider the case of $0<n_{0}<1$, $n\left(x\right)$ is an increasing function, therefore *p* is positive, and we should choose the sign ‘+’ in Equation (A29). Finally, separating the variables in Equation (A29),

(A30) $$\frac{dx}{x_{G}}=\sqrt{3}\frac{dn}{\sqrt{1-3n^{2}+2n^{3}}},$$

and, integrating Equation (A30), we get

(A31) $$\frac{x}{x_{G}}=\sqrt{3}\int_{n_{0}}^{n}\frac{dn}{\sqrt{1-3n^{2}+2n^{3}}}.$$

For convenience, we introduce

(A32) $$m=1-n,\;\;m_{0}=1-n_{0},$$

and rewrite Equation (A31) as

(A33) $$\frac{x}{x_{G}}=\;\sqrt{3}\int_{m}^{m_{0}}\frac{dm}{m\sqrt{3-m}}.$$

Taking the integral in Equation (A33), we get

(A34) $$\frac{x}{x_{G}}=\log\left(K_{0}\frac{\sqrt{3}+\sqrt{3-m}}{\sqrt{3}-\sqrt{3-m}}\right),$$

where an auxiliary constant $K_{0}$ is defined as follows:

(A35) $$K_{0}=\frac{\sqrt{3}-\sqrt{3-m_{0}}}{\sqrt{3}+\sqrt{3-m_{0}}}.$$

*m* can be now found explicitly from Equation (A34):

(A36) $$m\left(x\right)=3\left[1-{\left(\frac{\exp\left(x/x_{G}\right)-K_{0}}{\exp\left(x/x_{G}\right)+K_{0}}\right)}^{2}\right],$$

or, in terms of $n\left(x\right)$, we arrive at

(A37) $$n\left(x\right)=3{\left(\frac{\exp\left(x/x_{G}\right)-K_{0}}{\exp\left(x/x_{G}\right)+K_{0}}\right)}^{2}-2.$$

That is identical with the solution (39), if we take into account that the definition (A35) is actually identical with Equation (40).

## Appendix F. Approximate Stationary Solution in the Logistic Growth-Diffusion Model (Model 3)

If $n_{0}≃1$, then $m_{0}=1-n_{0}$ is a small value: $m_{0}≃0$. An expansion of Equation (A35) then yields:

(A38) $$K_{0}≃\frac{m_{0}}{12},$$

so that $K_{0}$ is also a small value. We then identically rewrite Equation (A37) as

(A39) $$n\left(x\right)=3{\left(\frac{1-K_{0}\exp\left(-x/x_{G}\right)}{1+K_{0}\exp\left(-x/x_{G}\right)}\right)}^{2}-2,$$

and then expand Equation (A39) to get

(A40) $$n\left(x\right)≃1-m_{0}\exp\left(-x/x_{G}\right),$$

or, finally,

(A41) $$n\left(x\right)≃1-\left(1-n_{0}\right)\exp\left(-x/x_{G}\right).$$

The latter result is identical with Equation (42). Of course, the approximate solution (A41) could be also obtained much easier, by observing that when $n_{0}≃1$ (and, therefore, $n\left(x\right)≃1$), the initial nonlinear ODE (Equation (A23)) can be approximated by a linear ODE

(A42) $$n^{\prime \prime }+\frac{1}{x_{G}^{2}}\left(1-n\right)=0,$$

and the solution of this linear ODE can be immediately found, yielding again Equation (A41).
